# Leading practices for men to support women’s health leadership: A toolkit of resources to initiate change

**DOI:** 10.1177/08404704221119099

**Published:** 2022-09-03

**Authors:** Ivy Lynn Bourgeault, Ruth Decady, Justine Pascual, Billie Jane Hermosura

**Affiliations:** 1 6363 University of Ottawa, Ottawa, Ontario, Canada.

## Abstract

Men have a critically important role to play in supporting women from different backgrounds to move into leadership roles. Indeed, it is necessary work for those in positions of privilege to challenge processes that result in inequitable gender outcomes in health leadership. We present the resources that have been compiled into a toolkit for men to support more inclusive health leadership and transformative systemic change. A three-step process was undertaken to search, select, and curate leading evidence-informed practices. Three key clusters of resources in the toolkit address why men’s actions are necessary, what leading actions entail, and the importance of mentorship and sponsorship. Change will require more than shaping the individual attitudes and behaviours of men in leadership positions. Attention to gender and other forms of inequity need to be embedded into the structures, processes and outcomes of teams, organizations, and systems and evaluated for process.

## Introduction

A watershed moment in women’s health leadership literature and advocacy occurred in 2019 when global statistics reported that women make up over 70% of the health workforce, but fewer than 25% of those are in health leadership positions.^1^ These statistics corroborate the report’s title in which healthcare is *Delivered by Women* yet *Led by Men.* Although it is difficult to assess where health leadership in Canada is specifically positioned vis-à-vis international benchmarks, because leadership diversity is not measured in the Canadian health sector, it is recognized that gender-inequity exists. We know, for example, that there have only been eight women presidents of the Canadian Medical Association and only eight women who have been deans of medical schools in Canada.^2^

The leadership literature is replete with advice for women wishing to seek leadership positions.^3^ They are encouraged to *lean in,*^4^ despite evidence showing that women face unique gender-based barriers on their leadership journeys.^5^ While it is important to empower women to undertake the path toward leadership, men also have a critically important role to play in supporting women from different backgrounds to move into leadership roles. Indeed, it is necessary work for those in positions of privilege to recognize gender-based inequity in representation and challenge processes that result in inequitable outcomes in health leadership seen across health systems, including in Canada.

### Purpose

This paper is part of a larger *Empowering Women Leaders in Health* project that examined the leading practices leading to the increased participation, visibility, and advancement of women and gender diverse people^1^ in positions of health leadership. The project activities included capturing lessons learned along women’s leadership pathways, sharing these with aspiring women leaders, and creating a LEADS^6^-informed toolkit. In this paper, we present the resources that have been compiled into a companion toolkit for men to support more inclusive health leadership and transformative systemic change.

## Methods

A three-part methodological process was undertaken to *search*, *select*, and *curate* leading practices for men to support of women leadership in health into a toolkit.

### Search

The original source of resources began with a targeted search of published peer reviewed and grey literatures (dating from 2015 to 2021). The purpose was to collect leading practices for men to recognize and address the systemic gender-based barriers that women of diverse groups uniquely face in their leadership journeys.

Social media platforms provide vast amounts of information unfiltered by typical gendered organizational power structures embedded in the published and grey literatures.^7^ As a result, we augmented the literature with resources in the form of quotes, infographics, podcasts, TED talks, and YouTube videos shared on social media—especially Twitter, Facebook, and LinkedIn. Social media provides space for non-traditional sources to be amplified in support of health leadership diversity. Gathering resources from high profile commentators and searching relevant hashtags, such as #HeForShe, were undertaken to create an inventory of potential tools. These searches began in 2019 and were updated in 2020 and 2021.^2^

To augment quotes from social media, we undertook seven semi-structured interviews with men in health leadership positions reflecting on their journey in support of women’s leadership. The following questions were posed: is there a gender-based health leadership gap in Canada? Why is women’s leadership in healthcare important? What can men do to support women’s health leadership? Excerpts from these interviews expanded upon resources selected for inclusion in the toolkit.

### Selection

Acquiring sources from traditional academic and non-traditional social media sources can pose challenges in the selection process. To this end, the team developed clear inclusion and exclusion criteria. Sources that explicitly mentioned keywords such as “allyship,” “mentorship,” and “sponsorship” were shortlisted for inclusion. Where overlap existed across two or more sources, we undertook a comparative analysis to identify the strongest resources for inclusion. In several cases, the most relevant portions of longer sources were excerpted for inclusion to ensure a toolkit that was light on text but supported with definitions and links to references for further exploration. All the resources selected were verified by two or more curators on the team and reviewed by the team lead.

### Curation

Once a shortlist of resources was compiled, they were clustered thematically into the most frequent, relevant, and highest impact topics that emerged from the literature and social media sources. Although the variability in sources can be more difficult to synthesize, the variability can also be seen as a strength because different types of tools may resonate with different users of the toolkit. In addition to the toolkit being reviewed by several team members and project advisors, each of the seven interviewees were invited to review and provide suggestions for further refinement of the toolkit. Additional refinements have been made to the resources based on feedback received through various presentation of the toolkit to a range of mixed gender audiences.

### Key toolkit components/topics

The three key clusters of resources in the toolkit address why men’s actions are necessary, what are some of the leading actions that could be undertaken, and a specific focus on the importance of mentorship and sponsorship.

### Why are men’s actions necessary?

Should users of the toolkit not be fully convinced of the need for men to take a more active role in creating more supportive environments and processes for women leaders on their journey, the first part of the toolkit provides evidence for its necessity. The key findings from the 2019 WHO review^1^ highlight how there is indeed a gender-based leadership gap that these gaps are driven by stereotypes, discrimination, power imbalance, and privilege and that women’s disadvantage intersects with other identities which taken together leads to a loss of talent, innovative ideas, and knowledge. The findings from these studies are bolstered with quotes from prominent men in the Canadian health leadership community interviewed for the project (see Box 1).

**Box 1**

Andre Picard, Health Columnist with the *Globe and Mail* describes the gender leadership gap he sees in healthcare: “If you look at who works in healthcare, it’s dominated by women … but as you move up the ladder that disappears. It becomes predominantly male. And we know all the cultural, social and economic reasons for that, but it doesn’t make it acceptable.”

Graham Dickson, Research Advisor to the Canadian Health Leadership Network, argues “It is so that they [women] can see themselves and know that their perspectives, their viewpoint has at least been considered in the decisions being made. … “If you’re in a patriarchal system, designed in a patriarchal way, and run only by men, then I could see [women] really losing interest.”

Dennis Kendel, former CEO of the College of Physicians and Surgeons of Saskatchewan reflects on how he wished he had become more engaged in transformative efforts earlier in his career, “I regret my delayed blindness to the factors and forces that caused blatant gender imbalance on the boards and management teams on which I served. When I reflect on how few women were on those teams, I am embarrassed that I did not earlier raise questions about that imbalance and take action to alter it. I regret my delayed understanding of the privileges I accrued, not through merit, but by simply being male. I regret my delayed insight into the inevitable reality that “privilege begets privilege”. … When I belatedly gained awareness of these failings, I did become progressively more engaged in mentorship and ally roles with women. Those experiences proved to be among the most meaningful things I did during my career.”

This section of the toolkit also highlights how the inequity experienced by women leaders is not just an equity issue but is critical for good patient care. Articles, such as *How Discrimination Against Female Doctors Hurts Patients*,^8^ which summarizes evidence of how women physicians bring unique perspectives to their practice, engage in more preventative care, and more-effective doctor-patient communication that can improve care. The authors argue that “in light of this evidence, it is reasonable to conclude that any practice, bias, or treatment that keeps women from entering and advancing in medicine is actually denying patients opportunities to receive higher-quality care.”

Furthermore, this section of the toolkit includes articles, such as *How Men Can Become Better Allies to Women,*^9^ where the authors argue that without the support of men, progress toward ending the gender leadership gap is unlikely. This section leads to other sections of the toolkit by providing evidence in support of men’s deliberate engagement in gender inclusive leadership programs and how men can and indeed need to be involved.

### What should men’s actions entail?

Once convinced of the necessity for men’s actions, the toolkit outlines leading practices for men’s actions. A key step is to recognize that identifying as a man is a privileged position in all gender systems, and other intersection identities of white, settler, cis-gender, heterosexual, and non-disabled are also privileged positions. Supporting gender equity in health leadership involves someone from a privileged group actively engaged in an ongoing process of deconstructing their own privilege.^10^

A critical next step is to *recognize unconscious biased assumptions about leadership* being a predominant domain for those who identify as men, which can be a key obstacle for women’s leadership journeys.^11^ Indeed, getting noticed as a leader in the workplace has been shown to be more difficult for those who identify as women, even when speaking up with similar ideas, strongly suggestive of unconscious assumptions about gender and leadership.^12^ Included in the toolkit are links to implicit association tests which helps all to assess these unconscious biases.^13^

This section of the toolkit reinforces that allyship is not an identity that can be claimed nor bestowed upon individuals, but rather is a continuous commitment to learning, unlearning, actively listening, and acting to create supportive environments for women on their leadership journey.^14^ Various tips for men compiled from different sources are included in Box 2.^15,16,17^

**Box 2: Common themes consolidated from tips for men to support women’s leadership**

Self-educate. Understand your privilege.

Listen: Ask women with intersecting identities about their experiences.

Be aware and attend to who is included in conversations and to nonverbal cues about who appears silent or uncomfortable.

Focus on the intersections. Notice sexist, racists and exclusionary words and phrases.

Reject passivity.

Speak up to disrupt and call out bias when it occurs.

Implement protocols for feedback.

Another key component of this section of the toolkit is resources to help *recognize and stop microaggressions*. Microaggressions are defined for toolkit users as “brief, everyday exchanges that send denigrating messages to certain individuals because of their group membership.”^18^ These can be “subtle snubs or dismissive looks, gestures and tones,”^15^ and can be related to gender, race, Indigenous status, sexuality, ability, and other forms of systemic inequities. The subtlety of some microaggressions can make them difficult to identify and address.

One particular microaggression delved into is *mansplaining,* which is defined by UN women^19^ as “the practice of a man explaining something to a woman in a way that shows he thinks he knows and understands more than she does.” Although it may not be intended to cause harm, it is describes as “a patriarchal act that trains women in self-doubt and self-limitation just as it exercises men’s unsupported overconfidence.” The toolkit includes specific examples of microaggressions and how to intervene as a bystander adapted from resources such as, *Eleven Things Not to Say to Your Female Colleagues*.^20^

## Moving from mentorship to sponsorship

Mentorship is often referred to as an important component of leadership development. The toolkit draws upon the following definition of mentorship as a “process in which a more skilled or more experienced person, serving as a role model, teaches, sponsors, encourages, counsels, and befriends a less skilled or less experienced person for the purpose of promoting the latter’s professional and/or personal development. Mentoring functions are carried out within the context of an ongoing, caring relationship.”^21^

We acknowledge the inherent power functions within mentorship relationships for toolkit users, and encourage awareness of the power dynamics and how these can impact the mentorship experience. Indeed, there can be a fine line between mentorship and mansplaining of which mentors should be aware. A *critical mentor* will thus be a supported and self-reflective person, who is aware that mentorship may be co-opted to serve aims other than that of the people within the mentorship relationship. The toolkit encourages men to take a critical approach to mentoring for gender inclusion.^22^

The toolkit also highlights how mentorship can be a two-way relationship where mentors can learn from their mentees as much as the other way around. *Reverse Mentoring* is a way for mentoring to help chip away at biases in both directions: Mentors come to believe that their protégés merit opportunities and in turn their sponsorship helps give protégés the breaks they need to develop and advance.^23^ *Push-Pull Mentoring* is another concept which consists of promoting people ahead of you while pulling people behind you.^24^ An example of push-pull mentoring is offered by Dr. Dennis Kendel, former CEO of the College of Physicians and Surgeons of Saskatchewan, who suggests: “When stepping away from board or senior management roles, men ought to reach out to women who may be interested in those roles, encourage them to apply and offer to serve as their ally.”

As noted in the definition of mentorship above, sponsorship in an integral but more active component; sponsorship involves being a kind of guarantor of the person you are sponsoring.^18^ As Bill Tholl, Founding Executive Director of the Canadian Health Leadership Network, details in the toolkit: “It is necessary but not sufficient to mentor those that are coming behind you but also sponsor them. The distinction there is to not just point to doors, but to actually help doors open.” In essence, sponsorship entails the sharing of the social capital represented by one’s networks developed over time as a leader with those for being sponsored.

Fear of reprisal may pose a barrier to men becoming mentors and sponsors for women on their leadership journey. Indeed, in a study of men’s engagement in gender-equity initiatives, nearly three quarters cited fear as a barrier to support gender equity.^25^ An element influencing this fear of engaging in a mentorship relationship can be the fear of being accused of inappropriate conduct. This fear can sideline conversations about the serious consequences for women of limited mentorship opportunities which can threatens to halt progress toward gender equity in leadership roles (see Box 3).^26^

**Box 3: Being an active bystander in the era of #MeToo and #TimesUp**

A bystander is a person who is present or hears about an incident but is not directly involved. An active bystander is one who acts. To be an active bystander, one can:

Diffuse - Make a lighthearted comment to try to stop the situation.

• Example: Sorry, what was that you said about women? What decade are you living in?

Call out - Calmly disagree and publicly declare the action or statement of the perpetrator to be wrong or unacceptable.

• Example: You might have thought it was just a joke, but I think that kind of comment is offensive.

Check in - Express your disapproval and ask if the target is ok.

• Example: That was so wrong of Sam to talk about your shirt like that. Are you OK? Do you want me to find out how you can report that kind of sexist behaviour?

Report - Access your organization’s sexual harassment reporting system and report details accurately.

• Example: Fatima visited the student union and asked for advice on how best to report an issue. She then lodged a written complaint.

Source: Take action: Empowering bystanders to act on sexist and sexually harassing behaviours.^27^

## Discussion

The resources compiled into the toolkit for men supporting women leaders help to increase awareness, offer leading practices for action, and are intended to encourage men on their leadership equity journey. It is but a start. Change will require more than shaping the individual behaviours of men, especially those in leadership positions, it will need to be augmented with data encouraging and measuring progress. As detailed in the LEADS framework,^6^ leadership starts with leading self but progresses to engaging others, achieving results, and developing coalitions for system transformation. Attention to gender and other forms of equity will need to be embedded into the structures, processes and outcomes of teams, organizations, networks, and systems to enable women from all backgrounds to achieve equity in leadership positions.

Achieving equity in health leadership requires dedicate resources to address equity outcomes and embed these processes within departments, divisions, and organizations. A critical first step is to assess the baseline status through environmental scanning and audits of equity data if they do not already exist. Consultation with a diverse range of interested stakeholders in high-level meetings to reach a consensus direction of equity initiatives aligned with organizational vision and values is another leading practice. Importantly, these equity plans will need to be adequately resourced to succeed. Continuous monitoring, reassessment, and evaluation to track progress toward equity goals and resetting direction for continuous improvement will need to be embedded in organizational processes for optimal results.^28^ Embedding these evaluative approaches may help to address the egregious lack of leadership diversity data in Canada noted at the outset.

A recent systematic review and meta-synthesis of organizational interventions to advance women in health leadership found that potentially effective interventions consistently reported that organizational leadership, commitment, and accountability were key drivers of organizational change.^29^ Health organizations, like Victoria Health in Australia, have developed resources to help organizations embed leadership equity initiatives starting with leadership, clear and enforced policy, laying best practice foundations, and tracking and reporting.^27^ Categories of leading organizational interventions included leadership development, awareness and engagement, mentoring and networking, organizational processes, and support tools.^27^ The tools we have curated in this toolkit are one key piece of the organizational change puzzle.

In addition to the need to expand the focus to the broader achieve results, develop coalitions, and system transformation elements of the LEADS framework, it is important to recognize that there is a difference between the develop of an evidence-informed toolkit and its deployment. The team partnered with the Canadian College of Health Leaders to embed the resources in broader leadership development. It complements the toolkit targeting women both embarking on a leadership journey and supporting other women’s leadership journeys as one of our interviewees mentioned *She-for-She.* Toolkits can have a fleeting shelf life and the commitment to refresh the resources on a yearly basis in partnership with the Ottawa Research Chair in Gender, Diversity, and the Professions is a promising approach.

## Notes

1. Although the project had an inclusive focus on women and gender diverse individuals from diverse racialized backgrounds, the focus of this toolkit was primarily on women; a toolkit in support of diverse leadership was curated into a separate toolkit which is written about in a separate manuscript.

2. A 2022 updated is in progress.
